# Amiodarone-Induced Cirrhosis of Liver: What Predicts Mortality?

**DOI:** 10.1155/2013/617943

**Published:** 2013-03-14

**Authors:** Nasir Hussain, Anirban Bhattacharyya, Suartcha Prueksaritanond

**Affiliations:** Department of Internal Medicine, Saint Joseph Hospital, Resurrection Health Care, 2900 North Lake Shore Drive, Chicago, IL 60657, USA

## Abstract

*Introduction*. Amiodarone has been used for more than 5 decades for the treatment of various tachyarrhythmias and previously for the treatment of refractory angina. There are multiple well-established side effects of amiodarone. However, amiodarone-induced cirrhosis (AIC) of liver is an underrecognized complication. *Methods*. A systematic search of Medline from January 1970 to November 2012 by using the following terms, amiodarone and cirrhosis, identified 37 reported cases of which 30 were used in this analysis. Patients were divided into 2 subsets, survivors versus nonsurvivors, at 5 months. *Results*. Aspartate aminotransferase was significantly lower (*P* = 0.03) in patients who survived at 5-months (mean 103.33 IU/L) compared to nonsurvivors (mean 216.88 IU/L). There was no statistical difference in the levels of prothrombin time, total bilirubin, alanine aminotransferase, alkaline phosphatase, gamma-glutamyl transpeptidase, cumulative dose, and latency period between the two groups. The prevalence of DM, HTN, HLD, CAD, and CHF was similar in the two groups. None of the above-mentioned variables could be identified as a predictor of survival at 5 months. *Conclusion*. AIC carries a mortality risk of 60% at 5 months once the diagnosis is established. Further prospective studies are needed to identify predictors of AIC and of mortality or survival in cases of AIC.

## 1. Introduction

Amiodarone has been used since the 1960s for the management of various tachyarrhythmias and in the past for refractory angina. There are multiple reported and well-established side effects of amiodarone therapy such as effects on the thyroid, skin, lungs, nerves, and cornea.

The effect of amiodarone on the liver resulting in hepatotoxicity is a recognized complication of amiodarone, but this hepatotoxicity leading to cirrhosis of the liver is unfortunately an underrecognized side effect. Little has been written on amiodarone-induced cirrhosis (AIC) of the liver due to its rarity [1–4]. The purpose of this paper is to review what we know so far about AIC of the liver.

## 2. Materials and Methods

### 2.1. Selection of Studies

A systematic search of Medline from January 1970 to November 2012 by using the following terms, amiodarone and cirrhosis, was performed; 37 reported cases were identified (Table 1) of which 30 were used in this analysis. We also searched the reference lists of all reported cases to identify citations that were not identified during the initial search. Data that were extracted for each patient included age, gender, latency period in years, whether ≥200 mg/day amiodarone dosage was used (it is thought that low-dose amiodarone has lesser side effects), cumulative dose, presence or absence of hypertension (HTN), diabetes (DM), hyperlipidemia (HLD), coronary artery disease (CAD), congestive heart failure (CHF), values of bilirubin, aspartate transaminase (AST), alkaline phosphatase (ALK P), albumin, alanine transaminase (ALT), and prothrombin time. The outcomes in terms of survival or mortality at 5 months were used. This specific timeline was selected because more than 50% of patients in these case reports died within 5 months after the diagnosis was established. Not all of the studies we found had all these data; however, they were included if the outcome was provided. In some cases, the outcome was not available, authors were contacted, and the outcome was determined. Those cases, for which outcome could not be determined, were excluded from the final analysis. Of seven cases that were excluded from the final analysis, one case had indeterminate cause of cirrhosis and for the rest of cases, outcome could not be established. Some authors provided cumulative doses for amiodarone. If total cumulative dose was not given, a formula (cumulative dose in grams = 365 ∗ no. of years ∗ total daily dose/1000) was used to estimate the final cumulative doses, with an assumption that the patient had been 100% compliant with the medicine. 

Prothrombin time for some cases was given as percentage activity rather than in seconds. We could not find any convertor for percentage activity to seconds (which is a normally reported unit). For cases in which prothrombin time was reported as a percentage prothrombin activity, values were not used in this analysis. 

### 2.2. Statistical Analysis

Of 37 cases analyzed, outcome was available only for 31 cases. One case had indeterminate cause of cirrhosis and was excluded, and 30 cases were used for our analysis. 

Survival at 5 months determined the patient subset and common pathophysiologic factors like DM, HTN, HLD, CAD, and CHF; lab values comprising of prothrombin time, total bilirubin, AST, ALT, ALK P, and GGT were compared between these two subsets. Logistic regression was used for comparing continuous independent variables, and chi-square test was used for comparing categorical variables. When the expected frequency was less than five, Fisher's exact test was used for categorical variables. Cox proportional hazards ratios were employed in determining prognosis. All statistical tests were two tailed with significance set at 95% level (*P* < 0.05). STATA 11 IC software was used for statistical analysis.

## 3. Results

AST was significantly lower (*P* = 0.03) in patients who survived at 5 months (mean 103.33 IU/L) compared to nonsurvivors (mean 216.88 IU/L). AST levels overall were raised in both groups and ranged between 64 IU/L and 734 IU/L (Tables 2 and 3). There was no significant difference in the levels of prothrombin time, total bilirubin, ALT, ALK P, and GGT between the two groups. The prevalence of DM, HTN, HLD, CAD, and CHF was similar in these two groups. Mean cumulative dose in cases of AIC was 280 g with a median latency period of 2.92 years and was statistically nonsignificant.

The risk of dying at 5 months was marginally higher in patients with high aspartate aminotransferase. Hazard ratio for death is 1.003 (95% CI ranging between 1.001 and 1.006). This finding was statistically significant (*P* = 0.03) (Table 3). Prothrombin time, total bilirubin, ALT, ALK P, GGT, and coexistence of DM, HTN, HLD, CAD, and CHF did not predict survival. Results are summarized in Tables 2 and 3.

Median age at the time of diagnosis of AIC was 68 years for all cases, 69.5 among survivors, and 67.5 among nonsurvivors. Most cases of AIC were observed among females though most who died were males.

## 4. Discussion

### 4.1. Pathogenesis of AIC

Amiodarone is a lipophilic agent [5] and tends to accumulate in lipid-laden organelles such as the liver. Amiodarone causes liver damage by different pathogenic mechanisms, one of which may include phospholipidosis. There are two different mechanisms by which amiodarone causes phospholipidosis.

 (I) Amiodarone and its metabolites (N-desmethylamiodarone) accumulate in lysosomes of hepatocytes, bile duct epithelium, and kupffer cells and leads to inhibition of phospholipase A1 and A2 [35, 36], which thereby inhibits removal of lysosomal lipids and leads to phospholipidosis.
(II) Amiodarone binds to phospholipids in lysosomes and forms a nondigestible complex [37, 38], which leads to phospholipidosis.

The exact mechanism of phospholipidosis-induced liver damage is unclear. Phospholipidosis has been reported to occur within two months of starting amiodarone therapy [6, 7]. Phospholipidosis occurs in a much larger percentage of patients receiving amiodarone [5] than actual hepatocellular damage (1% to 3%), which suggests that phospholipidosis may or may not have a role in the pathogenesis of AIC. In fact, development of phospholipidosis is considered a marker for accumulation of amiodarone [4] rather than a marker of hepatotoxicity.

Leakage [3, 4, 39, 40] of proteolytic enzymes from abnormal lysosomes represents another pathogenic mechanism of amiodarone-induced liver damage. Leakage of proteolytic enzymes may contribute to the elevation of aminotransferases and may over time lead to hepatic necrosis, fibrosis, and eventually cirrhosis. 

Immunologic mechanisms may be involved in pathogenesis in instances of amiodarone-induced acute hepatitis in patients with positive Coombs' test [41]. 

Amiodarone-induced inhibition of cellular respiration is another possible pathogenic mechanism for amiodarone-induced liver damage. Impairment of mitochondrial *β*-oxidation and uncoupling of oxidative phosphorylation leads to the formation of reactive oxygen species, which in turn has a role in the development of AIC [42–44].

### 4.2. Pathology

On histologic examination of biopsy samples obtained from amiodarone-induced cirrhotic patients; leukocytic infiltrate and strikingly high Mallory's hyaline along with other usual pathologic findings of cirrhosis are noted. High Mallory's hyaline or Mallory's bodies are suggestive of AIC. 

Mallory's hyaline is an eosinophilic inclusion made up of intermediate keratin filaments. Mallory's hyaline is not specific for AIC and may be seen in primary biliary cirrhosis, alcoholic cirrhosis or hepatitis, nonalcoholic cirrhosis, hepatocellular cancer, morbid obesity, and some other conditions. Mallory hyaline in AIC is present in zone 1 of acinus, whereas in alcoholic liver disease they are located usually in zone 3 [4]. 

Histologic findings in patients with amiodarone-induced hepatic damage are similar to those caused by alcohol [4, 45]. The complete pathologic spectrum of alcoholic like liver injury due to amiodarone includes micro- and macrovesicular steatosis, steatonecrosis, mega mitochondria, portal inflammation, fibrosis, and cirrhosis. Amiodarone-associated epithelloid granulomas have also been reported [8]. 

Presence of phospholipids laden lamellar lysosomal inclusion bodies on electron microscopy [1, 4, 37] is another characteristic pathologic finding of AIC of liver. Yap et al. observed that amiodarone-induced lysosomal inclusions developed in nearly 100% of patients at a period of 2 weeks [46].

### 4.3. Diagnostic Workup

AIC is a diagnosis of exclusion. Extensive workups are normally done to exclude other diagnoses including viral etiology, Wilson's disease, hemochromatosis, alpha 1 antitrypsin deficiency, alcoholic hepatitis, congestive liver damage, autoimmune liver pathologies, and hepatitis due to other drugs and toxins. There is no specific diagnostic lab test for AIC of the liver. 

Furthermore, there is no specific imaging characteristic for AIC of the liver although sometimes increased liver density may be noted on a noncontract CT scan of liver. Increased liver density is thought to be secondary to increased iodine content in the liver. Amiodarone has two atoms of iodine that constitute 37% of molecular weight of the drug. Enhanced density due to amiodarone is reversible upon discontinuation of amiodarone [47]. 

Diagnosis of AIC is usually based on liver biopsy. Mallory hyaline and lamellar lysosomal inclusions are typical of amiodarone-induced liver damage. 

### 4.4. Presentation

Amiodarone-induced liver damage may present as Reye's syndrome in kids [9] and may present as asymptomatic elevation of liver enzymes in adults. Asymptomatic liver enzyme elevation occurs in 25% of the population treated with amiodarone [1] and is usually reversible upon discontinuation of therapy [45]. Normalization of liver enzymes may take place anywhere from three weeks to nine months [48]. Symptomatic hepatic dysfunction occurs in less than 1% of the population treated with amiodarone and includes acute and chronic liver injuries. Acute liver injury includes acute hepatitis (idiosyncratic reaction may be involved in pathogenesis), whereas chronic liver injury includes steatosis (macro and microvesicular steatosis) and cirrhosis.

## 5. General Discussion

Amiodarone is an iodinated benzofuran derivative, lipophilic drug with a half-life of 35–110 days and a very large volume of distribution (VD). Amiodarone comes as number eight in drugs that cause drug-related hepatic fatalities [9]. Major metabolite of amiodarone, N-desmethylamiodarone, is not only pharmacologically active but has a longer elimination half-life and a larger VD than the parent drug [1, 10, 49–51]. Amiodarone and N-desmethylamiodarone may be detected even months after stopping the drug [51] as amiodarone accumulates in lipid reservoirs and is released slowly from these reservoirs. Due to this storage mechanism, amiodarone concentration in liver may be as high as 500-fold of serum level [52]. Based on these facts, damaging effects of amiodarone may persist up to one year after complete discontinuation of therapy [1]. Since amiodarone is mainly metabolized in the liver, any damage to the liver from any cause would hamper amiodarone metabolism and lead to a vicious cycle of accumulation of amiodarone and further amiodarone-induced hepatic damage [53].

Why some patients develop cirrhosis or hepatic damage as a side effect of amiodarone is not entirely clear. It has been suggested that differing sensitivity to amiodarone toxicity in population may exist [54]. Most patients who developed AIC usually used amiodarone PO 200 mg or more per day for more than 1-2 years. In light of the above-mentioned fact, researchers propose that the total cumulative dose of amiodarone may be important in estimating the risk of irreversible liver injury [55]. A cumulative dose of 380 g is suggested to associate with hepatotoxicity leading to cirrhosis [50]. Other prospective studies showed that amiodarone hepatic toxicity correlates to steady state serum levels of amiodarone rather than daily or cumulative doses [54, 56–58]. For example, if daily dosage of amiodarone during long-term therapy is reduced, despite increasing lifetime cumulative dose, the steady state serum concentration will still be reduced and thus decreasing risk of hepatotoxicity from amiodarone. Researchers have suggested that amiodarone level less than 1.5 mg/L has a minimal risk of hepatotoxicity, whereas a level above 2.5 mg/L may have a risk up to 6% for hepatotoxicity [5]. Patients with lower eject fraction may be more prone to hepatotoxic effects of amiodarone as suggested by Tisdale et al. [59]. Although it seems logical that patients with preexisting liver damage may be more prone to amiodarone-induced AIC of the liver, results by Kum et al. suggest otherwise [60]. 

 Besides chronic liver injury due to prolonged amiodarone use, acute hepatic side effects from amiodarone intravenous loading dose have been reported and are thought to be caused by polysorbate 80; a solvent used in drug preparation. This form of acute hepatotoxicity usually improves with discontinuation of medication, although fatalities have been reported [61]. 

To prevent amiodarone-induced cirrhosis, the amiodarone should be titrated to lowest effective dose. Patients should have baseline LFTs and then periodic monitoring of LFTs while on amiodarone (at 1, 3, and 6 months and then semiannually) [62]. Studies have suggested that baseline LFTs monitoring is performed only in 44% of patients, and a follow-up testing at 6 months and 1 year is done in only 41% and 35% of patients, respectively [63]. Patients found to have asymptomatic elevation of transaminases while on amiodarone should have a thorough investigation of the cause [56]; repeated testing may be necessary before labeling diagnosis of amiodarone-induced hepatotoxicity [45]. According to some authors, discontinuation of amiodarone for liver-related toxicity may not be necessary [57]. Kum et al. noted in their study that 50% of patients with increased transaminases while on amiodarone did not improve even after 1.5 years of drug withdrawal [60]. However, most authors suggest that if aminotransferases are two times above baseline value or above three times the upper normal limit, then amiodarone either should be reduced or discontinued [58, 61, 64, 65]. Withdrawal of drug, when irreversible liver damage has already occurred, has a very little effect on restoration of liver function. Despite what is said, discontinuation of amiodarone for liver-related hepatotoxicity may not be necessary except in cases to prevent irreversible loss of liver function [11]. After discontinuation of amiodarone, monitoring period should continue for at least one year as the damaging effect of amiodarone might persist. Patients on amiodarone should be advised to avoid any potentially hepatotoxic agent to prevent additive hepatic damage. Discontinuation of amiodarone is reported to occur in nearly 20–40% of patients if changes in aminotransferases are detected during amiodarone therapy [66]. 

 To date, the role of routine monitoring of amiodarone or its metabolite's serum levels for predicting hepatic damage is not well established. Nonetheless, there are numerous patients who may develop AIC without any abnormality in liver enzymes. Such patient population will not be detected until very late. Routine imaging may have a role in this subset of patients, but there is no study to support this. 

Based on our review (Table 1), we noted that AIC is extremely rare. However, once the diagnosis is established, the mortality risk may be as high as 60% at 5 months. The most common cause of death among reported cases was due to liver- and GI-related complications. Ethnic predisposition to AIC could not be determined due to lack of the published literature. The most common symptoms reported were generalized weakness, abdominal pain, and abdominal distension. 

A cumulative dose of 380 g has been suggested to associate with cirrhosis. By reviewing published cases, we realize that cirrhosis has been reported to occur with a cumulative dose as low as 55 g [9]. Why some patients with a cumulative dose of 200 g develop cirrhosis and others do not until cumulative dose crosses above 1000 g is unclear, but it does suggest that it may be a steady state concentration rather than cumulative dose that may be important in predicting risk of cirrhosis. The diagnosis in most patients was made by liver biopsy. Although Mallory hyaline and lysosomal inclusions are characteristic features of amiodarone-induced cirrhosis, the dilemma is that the above-mentioned pathologic characteristics may be identified in patients on amiodarone who do not have cirrhosis. Liver biopsy alone for diagnosis of AIC may not be enough. In fact, a thorough evaluation for all possible causes of cirrhosis should be done. Once all possible causes of cirrhosis have been ruled out, then only the presence of Mallory hyaline and lysosomal inclusions in cirrhotic liver may be suggestive of AIC of the liver. In all published cases, a thorough investigation for the cause of cirrhosis was carried out. In most published cases, obesity, DM, and ethanol consumption were given special attention to rule out the possibility of alcoholic and nonalcoholic steatohepatitis (NASH). Risk factors for development of amiodarone-induced hepatotoxicity and cirrhosis have not been clearly defined. Whether it is cumulative dose or steady state concentration of amiodarone that predicts risk of cirrhosis has not been determined. In most patients even after discontinuation of amiodarone, toxicity effects did not subside. Majority of patients have had amiodarone-related hepatotoxicity before onset of cirrhosis leading to a decrease in dose or discontinuation of the drug. However, the drug was restarted for various reasons, which unfortunately lead to cirrhosis. Although AST and ALTs are said to be mainly elevated in amiodarone-related hepatotoxicity and not the ALK P or GGT [56], our analysis suggests otherwise. According to our analysis, the only statistically significant variable different among survivors versus nonsurvivors at 5 months was the level of AST at time of diagnosis of AIC. We suggest that if patients on amiodarone have a persistent increase in aminotransferases, a liver imaging and a liver biopsy should be done. 

There is no treatment available for amiodarone-induced hepatotoxicity or cirrhosis besides discontinuation of the offending agent and switching to some other antiarrhythmic agent. Previously, antioxidants vitamin E and selenium have been tried without any success. We recommend against using antioxidants for counteracting amiodarone hepatotoxicity, as there is no strong scientific evidence for such practices. 

We are aware that there are limitations in our study because of the smaller sample size, which may have affected our analysis. Future prospective studies using larger patient population may be able to identify predictors of survival in case of AIC. 

## 6. Conclusion

Although amiodarone does have very serious and fatal effects on the liver, such effects are rare. With a closer monitoring and taking appropriate actions when prompted, amiodarone can be safely used on long-term basis. Further prospective studies are needed to identify predictors of AIC and of mortality or survival in cases of AIC. Role of routine imaging and biopsy of the liver in patients taking amiodarone is unclear; future studies are needed to address this issue.

## Figures and Tables

**Table 1:** 

*N*	Age Sex Race	PMH other than arrhythmias, drugs	Presentation Etoh use	Authors	Labs	Pathology	Duration and dosages	Outcome after diagnosis of AIC
1	63 M W	CAD s/p CABG, DM, HLD, No etoh, (amiodarone, warfarin, aspirin, rosiglitazone, and lovastatin) No other hepatotoxic drugs	Abdominal distension	Puli et al. [2]	23.94 114 ? 23 82	Micronodular cirrhosis, bridging fibrosis, lymphocytic infiltrate, macrophages, plasma cells, microvesicular steatosis, and lysosomal bodies on electron microscopy	600 mg/day for 10 days, then 200 mg/day for 22.5 months cumulative dose of approx. 141 g	Survived

2	81 F —	Digitoxin, alpha methyldopa No DM, and no etoh	Cirrhosis diagnosed during study	Guigui et al. [3]	15, 28 186, ?, 53 GGT 151, PT 76%	Portal fibrosis, steatosis, PMN, myelin figures, dense deposits, and cirrhosis	120 months, cumulative dosage of 520 g	Could not establish an outcome

3	72 M W	DM, HTN, and CKD No etoh, (amiodarone, simvastatin, and glipizide)	Ascites and fatigue	Atiq et al. [5]	32.49, 106, 147, ?, 75, PT 20	Steatohepatitis, Mallory hyaline, neutrophilic infiltrate, and cirrhosis	200 PO mg/day ∗ 3 years Cumulative dose of approx. 219 g	Died during the same admission due to complication of liver disease

4	67 F AA	CAD, CHF, and s/p AICD No etoh	Confusion	Atiq et al. [5]	? 377 551 ? 277 PT 12.7	Mallory hyaline, neutrophil infiltrate, pericellular/bridging fibrosis, and degenerating hepatocytes (cirrhosis found on autopsy)	Low dose ∗ 2 years	Died 2 weeks after the workup due to cardiopulmonary failure

5	63 M —	COPD, DM, and hypothyroidism (digoxin, furosemide, and thyroxin)	Abnormal LFTs on screening	Rigas et al. [6]	? 59 207 ? 28	Portal, central, and sinusoidal fibrosis and loss of lobular architecture and regenerative nodules, central vein sclerosis, Mallory bodies, lysosomal inclusion, and cirrhosis	400 mg/day Amiodarone ∗ 18 months Cumulative dose 216	No information about the outcome

6	73 M —	Heart failure, no significant etoh, complete right and left anterior hemiblock (translated through Google)	Jaundice, hepatomegaly	Capron-Chivrac et al. [7]	269 55 708 68 ? 224 GGT 781	Portal, periportal fibrosis, mixed inflammatory infiltrate, ductal proliferation, lysosomal inclusions, no Mallory bodies, and cirrhosis	Amiodarone 100 mg/day ∗ 5 days/wk ∗ 2 months Cumulative dose of 4 g “We strongly suspect in this case cirrhosis may have been due to some other cause”	Died 2 months after stopping amiodarone, died of pulmonary edema

7	70 F —	—	Weight loos and blurred vision	Chaabane et al. [8]	—	Micronodular cirrhosis, steatohepatitis	200 mg/day ∗ 15 years Cumulative dose of approx. 1095 g	Could not establish an outcome

8	62 M —	CAD, HTN, CHF, emphysema, pulmonary HTN, HLD, renal insufficiency, migraine, ulcerative colitis, cholelithiasis, and no etoh	Progressive weakness, abdominal discomfort, and jaundice	Anonymous [9]	51.3 734 119 29 781 PT 12.4	Micronodular cirrhosis with ballooning degeneration of hepatocytes and Mallory bodies, some steatosis	150–1000 mg/day, averaging 400 mg/day ∗ 8.5 years Cumulative dose of approx. 1241 g	Died 2 weeks after stopping medicine, probably hepatic encephalopathy

9	73 M —	Obesity, moderate alcohol intake	Fatigue, weakness	Anonymous [9]	34.2 ? 115 ? 67	Mallory bodies, minimal fatty change, and cirrhosis	Amiodarone 300 mg/day ∗ approx. 6 months Cumulative dose of approx. 55 g	Survived for more than 3 years

10	64 M —	Gout, CAD, and renal failure secondary to lead intoxication, MI, pulmonary edema, and sylvian microembolism (digoxin, warfarin, diclofenac, amiodarone, and allopurinol)	Fatigue, weight loss, 1-2 alcoholic beverages on social occasions	Richer and Robert [10]	12 149 176 ? 112 INR 2.4 GGT 277	Ballooned hepatocytes, Mallory bodies, fibrosis, phospholipidosis, inflammatory infiltrate, and cirrhosis	2.8 g ∗ 4 days, then amiodarone 400 mg–600/day ∗ 13 months, cumulative dose of approx. 206 g	Died 69 days after cessation of therapy, due to hepatorenal syndrome

11	74 M —	Ischemic heart disease, CVA, poliomyelitis, carotid endarterectomy, and peripheral neuropathy Etoh Consumption = negligible	Muscle weakness, hepatomegaly	Gilinsky et al. [11]	133.4 110 275 16 231 PT 17	Fibrosis, Mallory hyaline, lysosomal inclusions, amiodarone, and desmethylamiodarone conc. 0.6, 0.5 mg/L, respectively	Amiodarone 300–600 mg/day ∗ 28 months Cumulative dose of approx. 378 g	Died despite discontinuation of therapy, probably liver failure

12	76 F —	No significant past history other than recurrent SVTs	Abdominal pain, anorexia, and wasting	Tordjman et al. [12]	n 225 317 29 ?	Mallory bodies, fibrosis, severely damaged hepatocytes, bile duct proliferation, and cirrhosis	200 mg daily ∗ 5 years Cumulative dose od approx. 365 g	Died 2 weeks after evaluation due to hepatic encephalopathy

13	77 F —	—	Anorexia, abdominal pain, and malaise	Rene et al. [13]	20.52, 16 324, 39 10, GGT 66 PT 70%	Micronodular cirrhosis, central and periportal fibrosis, and probable phospholipidosis	400 mg/day ∗ 9 years Cumulative dose of approx. 1314 g	Outcome could not be determined

14	79 M —	CAD s/p CABG, HLD hypothyroidism, and s/p pacemaker (amiodarone, ASA, furosemide, atorvastatin, and ranitidine) <2 units etoh/month No herbal medicines	Upper GI bleed, lethargy ∗ 2 months	Singhal et al. [14]	14 67 216 27 GGT 443	PMN infiltrate, reduplicating bile ducts in hepatic nodules, degenerating hepatocytes, Mallory bodies, extensive fibrosis, and cirrhosis	200 PO mg/ day ∗ 33 months Cumulative dose of approx. 198 g	Died 3 months after diagnosis despite of stopping amiodarone due to heart and renal failure and hepatic encephalopathy

15	75 F —	s/p MI, left ventricular aneurysm, and normal coronaries	Abnormal LFTs —	Bach et al. [15]	? 140 850 ? ?	Micronodular cirrhosis, portal fibrosis, Mallory bodies, ballooning hepatocytes, phospholipidosis, inflammatory cells, and lysosomal inclusions	800 mg/day ∗ 7 months, then 600 mg/day ∗ 24 months, then 200 mg/day for 3 months Cumulative dose of approx. 624 g	Survived for more than 3 years

16	63 F —	Mitral valve stenosis s/p replacement 5 years ago, moderate TR, no obesity, and no diabetes (amiodarone, coumadin derivative)	Asthenia, anorexia, and weight loss of 8 kg for 5 months	Martinez et al. [16]	18.8 198 301 35 86, GGT 475 PT 20%	Postmortem liver biopsy showed incipient cirrhosis, portal fibrosis, inflammatory ductal infiltration and mixed leukocytic infiltration, steatosis, Mallory bodies, and acidophilic change	400 mg daily ∗ 5 days/week, duration not specified, may be >12 years Cumulative dose of approx. >1152 g	Died due to massive upper GI bleed during same admission

17	58 M —	CAD, MI (amiodarone, aspirin, furosemide, diltiazem, isosorbide dinitrate, digoxin, famotidine) No etoh, no herbs used	Abdominal distension and fatigue	Çoban et al. [17]	? 64 ? 11 ? PT 16.9 GGT 133	Polymorph nuclear infiltrate, ductal proliferation, fibrosis, bridging necrosis, vacuolar degeneration, lysosomal inclusions (73+), and cirrhosis	200 mg daily ∗ 1 year, stopped due to side effects, restarted 200 mg daily ∗ 6 years Cumulative dose of approx. 511 g	Died 3 months after diagnosis due to hepatorenal syndrome and hepatic encephalopathy

18	85 M —	Ischemic heart disease No obesity, no etoh, and no DM	Cardiac congestion	Oikawa et al. [18]	20.52 81 452 ? 35 GGT 210 PT 59%	Polymorph nuclear infiltrate, ductal proliferation, fibrosis, micro/macrovesicular steatosis, lysosomal inclusions, and cirrhosis (cumulative dose given by author = 528)	400 mg daily ∗ 17 days, then 200 mg daily for 84 months Cumulative dose of approx. 518 g	Died 5 months after diagnosis due to renal failure and pneumonia

19	49 M —	Rheumatic heart disease, endocarditis, HTN, and DM (amiodarone, acebutolol, and glibenclamide)	Pain in RUQ and fever	Lamproye et al. [19]	n n n n *5ULN PT n	Micronodular cirrhosis, portal fibrosis, leukocytic infiltrates, Mallory bodies, micro and macrovesicular steatosis	400 mg/day ∗ 5 days a week ∗ 12 years Cumulative dose of approx. 1152	No outcome given

20	56 F —	s/p pacemaker, goiter No DM, no obesity, and no etoh abuse (digoxin, acenocoumarin, and amiodarone )	—	Babany et al. [20]	15 38 73 ? 63 PT 100%	Micronodular cirrhosis, marked steatosis, inflammatory infiltrate, Mallory bodies, lysosomal inclusions (268 g cumulative dose given by author) Amiodarone and N-desmethylamiodarone plasma conc. 0.42, 0.70 g/L	400 mg/day ∗ 5 days per week 2 years, then 200 mg ∗ 5 days per week ∗ 11 months. Cumulative dose of approx. 236 g	Survived for more than 10 months

21	83 F —	Angina pectoris No DM, no obesity, and no etoh abuse (amiodarone )	Hepatomegaly	Babany et al. [20]	15 63 139 ? 139 PT 100%, GGT 460	Fibrosis, steatosis, Mallory bodies, inflammatory infiltrate, cirrhosis, and lysosomal inclusions on electron microscopy (220 g cumulative dose given by author)	Amiodarone 200 mg/day ∗ 3.5 years Cumulative dose of approx. 256 g	Survived for more than 1.5 years

22	68 F —	Commissurotomy for mitral stenosis No DM, No obesity, No etoh abuse (hydroxyzine, amiodarone, tiodomarol, and clonazepam)	Abnormal LFTs	Babany et al. [20]	11 43 88 ? 60 PT 100%	Moderate fibrosis, steatosis, polymorph nuclear infiltrate, Mallory bodies, cirrhosis, and lysosomal inclusions (211 g cumulative dose given by author)	Amiodarone 200 mg daily ∗ 5 days/week for 3 years, then 100 mg/day for 2 years, and then 200 mg/day ∗ 6 months Cumulative dose approx. = 254 g	Survived at least more than 9 months

23	68 M —	No sig. PMH (isosorbide, warfarin) No etoh	No significant history besides arrhythmia	Rinder et al. [21]	? 180 422 ? ?	Active cirrhosis, ongoing hepatocytes destruction, and Mallory bodies (165 g given by author)	Loading dose for 1 month, then 400 mg/day ∗ 13.5 months. Cumulative dose of approx. 162 g	Died one month after discontinuation of drug due to hepatic encephalopathy and hepatorenal syndrome

24	64 F —	WPW syndrome, no significant PMH (amiodarone, diuretic, and beta blocker) No etoh used	Weakness, bedridden, and ascites	Shepherd et al. [22]	44 172 150 ? ?	Micronodular cirrhosis with extensive necrosis of regenerating nodules, fibrosis, and swollen hepatocytes	600 mg/day ∗ 4 years Cumulative dose of approx. 876 g	Died due to bronchopneumonia, diagnosis of cirrhosis made at postmortem

25	76 M —	Ischemic heart disease, pulmonary edema (amiodarone, isosorbide dinitrate, furosemide, and potassium) No etoh	—	Jeyamalar et al. [23]	? 66 148 35 69 PT normal	Moderate inflammatory cells, nodules enclosed in fibrous bands, fatty, bile ductules proliferation change, and early cirrhosis Total cumulative dose 215 g	600 mg/day ∗ 1 week, 400 mg/day ∗ 1 month, and 200 mg ∗ 5 days/week ∗ 4 years Estimated dose = 208.2 g	Survived for more than 4 years

26	67 M —	Hypertrophic obstructive cardiomyopathy, s/p ICD, no obesity, no etoh, and no DM	B/L hand tremor	Ishida et al. [24]	32.49 88 463 ? 167 PT 17.4	Micronodular cirrhosis, swollen hepatocytes, proliferating bile ductules, inflammatory infiltrate, micro/macrovesicular steatosis, Mallory bodies, and lysosomal inclusion bodies Calculated dose comes out to 158	200 mg/day ∗ 26 months Cumulative dose of 206 g	Died 8 days after admission due to prerenal failure

27	57 M —	MI	Lethargy, abdominal distension	Harrison and Elias [25]	—	Micronodular cirrhosis, proliferating bile ducts, neutrophil infiltrate, Ballooning degeneration of hepatocytes, Mallory hyaline, lysosomal inclusion bodies, and epithelioid granulomas	200 mg twice/day ∗ 4 years 6 months Cumulative dose of approx. 657 g	Needed liver transplant

28	77 F —	HTN, DM, hypothyroidism, and GERD (Lisinopril, glimepiride, esomeprazole, levothyroxine, amiodarone, furosemide, spironolactone, propranolol, and isosorbide dinitrate) No obesity, no alcohol	New onset ascites and variceal hemorrhage Abdominal distension, lower extremity swelling, and SOB	Raja et al. [26]	10.26 54 216 39 32 INR 1.2 GGT 230	Lymphocytic infiltration, macro/microvesicular steatosis, Mallory hyaline, ballooning degeneration, pericellular fibrosis, cirrhosis, and bridging fibrous septa	Amiodarone 200 mg/day ∗ 3 years Cumulative dose of approx. 219 g (No herbal medicines)	Survived more than 6 months confirmed with author

29	77 M —	CAD, s/p MI, s/p CABG, hep. B infection, and CHF No etoh for last 14 years (serum amiodarone and N-desmethylamiodarone levels = 3 & 2.6 mg/L)	Fatigue, weight loss, and abdominal swelling	Flaharty et al. [27]	46.17 223 459 30 124 PT 39 GGT 738	Marked fibrosis, inflammatory infiltrate Mallory bodies, cirrhosis, proliferating ductules, and lysosomal inclusions Cumulative dose of 202 g (given by author)	1200 mg ∗ 13 days, 400–600 mg/day ∗ 12 months Cumulative dose of approx. 200 g	Died on day 21 of hospitalization due to bradycardia episode

30	62 F W	No etoh	Weakness and jaundice	Snir et al. [28]	92.3, 520 329, 22 131 GGT 1493	Micronodular cirrhosis, moderate to severe fibrosis of portal tract, pericellular fibrosis, Mallory bodies, and cirrhosis on postmortem	Amiodarone 800 mg/day dose ∗ 1 year Estimated cumulative dose = 292 g	Died due to liver failure 3 weeks after stopping drug

31	84 F W	HTN, CHF, obesity (amiodarone, felodipine, furosemide, potassium supplement, aspirin, and cisapride) No other drugs No etoh	Dark brown urine for 7 days	Chang et al. [29]	142 130 610 30 50	Portal fibrosis, pericellular sinusoidal fibrosis, lysosomal inclusions, Mallory bodies, and cirrhosis 1.3/1.3 amiodarone/ desmethylamiodarone serum levels	400 mg/day ∗ 5 years Cumulative dose = 730 g	Died 4 months after diagnosis despite of stopping drug, no cause of death established

32	72 M Korean	HTN (amiodarone, felodipine, HCTZ, and aspirin) No etoh EF 70%	Sudden onset abdominal distension	Sung and Yoon [30]	46.1 317 137 27 237 GGT 385 INR 1.32	Cirrhosis, polymorphnuclear infiltrate, Mallory bodies, ballooning degeneration, and lysosomal inclusions	Amiodarone 200 mg/day ∗ 5 years Estimated cumulativ dose = 365 g	Survived, amiodarone discontinued, and other antiarrhythmic started

33	64 F —	Unstable recurrent angina, ventricular aneurysm, and MI No etoh, no DM	For surgical resection of ventricular aneurysm	Poucell et al. [31]	12 ? 188 38 82 PT 11	Micronodular cirrhosis, Mallory bodies, ballooning, macrovesicular steatosis, fibrosis, inflammation, pleomorphic mitochondria, and lysosomal inclusion	600 mg ∗ 5 days/wk ∗ 2 years Cumulative dose of 288	Died shortly after liver biopsy, cause unknown may be MI, and no postmortem

34	62 M —	Etoh 85 g/day, s/p MI, CAD	Hepatomegaly despite normal LFTs ∗ 1 year, presented for liver biopsy	Poucell et al. [31]	8.5 ? 98 37 80 PT 11	Micro nodular cirrhosis, Mallory bodies, ballooning, macro vesicular steatosis, fibrosis, inflammation, pleomorphic mitochondria, lysosomal inclusion	600 mg ∗ 5 days/wk ∗ 2 years Cumulative dose 288 g	Continued on amiodarone and survived

35	70 M —	Alcoholic cardiomyopathy, adrenal insufficiency (amiodarone, hydrocortisone) Etoh was DC when heart problem diagnosed	Jaundice, pruritus, and deterioration of condition	Salti et al. [32]	481 95 1451 ? 92 GGT 1231	Portal and septal fibrosis, polymorph infiltrate, Mallory bodies, lysosomal inclusions, macrovesicular steatosis, and cirrhosis	Amiodarone 200 mg ∗ 5 days/wk ∗ 2 years Estimated cumulative dose of 96	Survived for more than 5 months at least

36	77 F —	A fib	Weakness, nausea, vomiting, abdominal distension, lethargy, and confusion	Chandraprakasam and Whitcraft [33]	? 192 122 ? 162	Neutrophilic satellitosis, Mallory hyaline, foam cells representing phospholipidosis, macrovesicular steatosis, and cirrhosis	Amiodarone 200 mg/day ∗ 4 years Cumulative dose = 292	No information about outcome

37	68 M —	Depression, heart failure (digoxin, doxepin, and bumetanide)	Vomiting and muscle weakness of one month duration	Lim et al. [34]	28 142 854 29 ?	—	400 mg/day ∗ 5 months, then 600 mg/day for 16 months Cumulative dose of approx. 348 g	Died due to liver failure 5 months after diagnosis despite of stopping amiodarone

*N*: number, DM: diabetes mellitus, HTN: hypertension, HLD: hyperlipidemia, CAD: coronary artery disease, CABG: coronary artery bypass grafting, SVT: supra ventricular tachycardia, CHF: congestive heart failure, etoh: alcohol, HCTZ: hydrochlorothiazide, ASA: aspirin, SOB: shortness of breath, n: normal, TR: tricuspid regurgitation, ULN: upper limit of normal, and W: white.

Labs are written in the following sequence, Bili, AST, ALK P, Albumin, and ALT in all tables. AST, ALT, ALK P, and GGT values are given in IU/L, bilirubin is given as Mmol: micromole/L (2–17) normal range, albumin is given as g/L.

*ULN stands for upper limits of the normal and the written lab is for ALT being 5 times the ULN.

**Table 2:** Characteristics of reported cases.

	All cases, *N* = 30	Survivors *N* = 12	Nonsurvivors at 5 months *N* = 18	*P*
Median age (years)	68 (56–85)	69.5 (56–83)	67.5 (58–85)	0.84
Male/female	18/12	7/5	11/7	1.00
Diabetes	*N* = 3	2	1	0.23
HTN	*N* = 7	2	5	1.00
Hyperlipidemia	*N* = 5	2	3	1.00
CAD	*N* = 16	5	11	0.26
CHF	*N* = 11	3	8	0.39
>200 mg/<200 mg Amiodarone dose given	18/12	5/7	13/5	0.33
Latency period	2.92 (0.5–12)	3.06 (0.5–5.5)	2.54 (1–12)	0.45
Cumulative dose	279.92 (55–1241)	279.92 (55–657)	465 (165–1241)	0.07
Prothrombin time	16.76 (11–39)	13.68 (11–17)	18.92 (11–39)	0.08
Bilirubin (micromole/liter)	55.73 (9–481)	71.67 (9–481)	46.16 (12–142)	0.53
AST	177.58 (64–734)	103.33 (38–317)	216.88 (64–734)	**0.03**
ALK P	352.96 (73–1451)	331.5 (73–1451)	365.59 (119–854)	0.78
Albumin	28 (11–39)	31 (23–39)	26.91 (11–38)	0.32
ALT	134.59 (32–781)	92.1 (32–237)	170 (35–781)	0.16
GGT	569 (133–1493)	640.33 (230–1231)	538.43 (133–1493)	0.74

*N* refers to the number of patients in each group. Not all studies had data on all variables.

Median values given for latency period, and for all other variables, mean values are given.

**Table 3:** Prognostic indicators of 5-month survival.

	Hazard ratio	95% Confidence interval	*P*
Age	0.99	(0.94–1.05)	0.80
Sex	0.90	(0.34–2.37)	0.83
Diabetes	0.40	(0.05–3.08)	0.32
HTN	1.34	(0.43–4.10)	0.62
Hyperlipidemia	1.09	(0.29–4.03)	0.90
CAD	1.49	(0.51–4.36)	0.46
CHF	1.74	(0.52–5.80)	0.36
>200 mg/ <200 mg Amiodarone dose given	1.35	(0.48–3.84)	0.56
Latency period	1.12	(0.90–1.38)	0.33
Cumulative dose	1.001	(0.999–1.003)	0.09
Prothrombin time	1.04	(0.97–1.11)	0.36
Bilirubin (micromole/liter)	0.998	(0.99–1.00)	0.45
AST	1.003	(1.001–1.006)	**0.03**
ALK P	1.000	(0.999–1.001)	0.98
Albumin	0.99	(0.91–1.09)	0.89
ALT	1.002	(0.999–1.005)	0.21
GGT	1.000	(0.998–1.002)	0.87

Not all studies had data on all the variables.

## References

[1] Lewis JH, Ranard RC, Caruso A (1989). Amiodarone hepatotoxicity: prevalence and clinicopathologic correlations among 104 patients. *Hepatology*.

[2] Puli SR, Fraley MA, Puli V, Kuperman AB, Alpert MA (2005). Hepatic cirrhosis caused by low-dose oral amiodarone therapy. *American Journal of the Medical Sciences*.

[3] Guigui B, Perrot S, Berry JP (1988). Amiodarone-induced hepatic phospholipidosis: a morphological alteration independent of pseudoalcoholic liver disease. *Hepatology*.

[4] Lewis JH, Mullick F, Ishak KG (1990). Histopathologic analysis of suspected amiodarone hepatotoxicity. *Human Pathology*.

[5] Atiq M, Davis JC, Lamps LW, Beland SS, Rose JE (2009). Amiodarone induced liver cirrhosis. Report of two cases. *Journal of Gastrointestinal and Liver Diseases*.

[6] Rigas B, Rosenfeld LE, Barwick KW (1986). Amiodarone hepatotoxicity. A clinicopathologic study of five patients. *Annals of Internal Medicine*.

[7] Capron-Chivrac D, Reix N, Quenum C, Capron JP (1985). Amiodarone hepatopathy. Study of a case and review of the literature. *Gastroenterologie Clinique et Biologique*.

[8] Chaabane NB, Hellara O, Safer L (2012). Cirrhosis with increased density of the liver: amiodarone-induced hepatotoxicity. *Tunisie Medicale*.

[9] http://livertox.nih.gov/Amiodarone.htm.

[10] Richer M, Robert S (1995). Fatal hepatotoxicity following oral administration of amiodarone. *Annals of Pharmacotherapy*.

[11] Gilinsky NH, Briscoe GW, Kuo CS (1988). Fatal amiodarone hepatoxicity. *American Journal of Gastroenterology*.

[12] Tordjman K, Katz I, Brusztyn M, Rosenthal T (1985). Amiodarone and the liver. *Annals of Internal Medicine*.

[13] Rene JM, Buenestado J, Pais B, Pinol MC (1995). Cirrosis causada por amiodarona. *Revista Espanola de Enfermedades Digestivas*.

[14] Singhal A, Ghosh P, Khan SA (2003). Low dose amiodarone causing pseudo-alcoholic cirrhosis. *Age and Ageing*.

[15] Bach N, Schultz BL, Cohen LB (1989). Amiodarone hepatotoxicity: progression from steatosis to cirrhosis. *Mount Sinai Journal of Medicine*.

[16] Martinez R, Saperas E, Vilaseca J, Tornos P, Allende H (1994). Cirrosis hepatica inducida por amiodarona. *Gastroenterologia y Hepatologia*.

[17] Çoban Ş, Köklü S, Sencer H, Ekiz F, Örmeci N (2007). Low dose amiodarone associated cirrhosis. *Journal of Gastroenterology and Hepatology*.

[18] Oikawa H, Maesawa C, Sato R (2005). Liver cirrhosis induced by long-term administration of a daily low dose of amiodarone: a case report. *World Journal of Gastroenterology*.

[19] Lamproye A, Ramos J, Larrey D, Belaiche J (1998). Pseudo-alcoholic hepatitis and cirrhosis caused by Amiodarone (Cordarone). *Revue Médicale de Liège*.

[20] Babany G, Mallat A, Zafrani ES (1986). Chronic liver disease after low daily doses of amiodarone. Report of three cases. *Journal of Hepatology*.

[21] Rinder HM, Love JC, Wexler R (1986). Amiodarone hepatotoxicity. *The New England Journal of Medicine*.

[22] Shepherd NA, Dawson AM, Crocker PR, Levison DA (1987). Granular cells as a marker of early amiodarone hepatotoxicity: a pathological and analytical study. *Journal of Clinical Pathology*.

[23] Jeyamalar R, Pathmanathan R, Wong D, Kannan P (1992). Hepatotoxicity of amiodarone. *Annals of the Academy of Medicine Singapore*.

[24] Ishida S, Sugino M, Hosokawa T (2010). Amiodarone-induced liver cirrhosis and parkinsonism: a case report. *Clinical Neuropathology*.

[25] Harrison RF, Elias E (1993). Amiodarone-associated cirrhosis with hepatic and lymph node granulomas. *Histopathology*.

[26] Raja K, Thung SN, Fiel MI, Chang C (2009). Drug-induced steatohepatitis leading to cirrhosis: long-term toxicity of amiodarone use. *Seminars in Liver Disease*.

[27] Flaharty KK, Chase SL, Yaghsezian HM, Rubin R (1989). Hepatotoxicity associated with amiodarone therapy. *Pharmacotherapy*.

[28] Snir Y, Pick N, Riesenberg K, Yanai-Inbar I, Zirkin H, Schlaeffer F (1995). Fatal hepatic failure due to prolonged amiodarone treatment. *Journal of Clinical Gastroenterology*.

[29] Chang CC, Petrelli M, Tomashefski JF, McCullough AJ (1999). Severe intrahepatic cholestasis caused by amiodarone toxicity after withdrawal of the drug: a case report and review of the literature. *Archives of Pathology and Laboratory Medicine*.

[30] Sung PS, Yoon SK (2012). Amiodarone hepatotoxicity. *Hepatology*.

[31] Poucell S, Ireton J, Valencia-Mayoral P (1984). Amiodarone-associated phospholipidosis and fibrosis of the liver. Light, immunohistochemical, and electron microscopic studies. *Gastroenterology*.

[32] Salti Z, Cloche P, Weber P, Houssemand G, Vollmer F (1989). A case of amiodarone-induced cholestatic hepatitis. *Annales de Cardiologie et d’Angeiologie*.

[33] Chandraprakasam S, Whitcraft M (2012). Pseudo-alcoholic cirrhosis: an interesting case of amiodarone induced hepatotoxicity. *The Internet Journal of Gastroenterology*.

[34] Lim PK, Trewby PN, Storey GCA, Holt DW (1984). Neuropathy and fatal hepatitis in a patient receiving amiodarone. *British Medical Journal*.

[35] Itostetler K, Reasor M, Frazee BW (1984). Mechanisms of amiodarone toxicity: inhibition of phospholipase A. *Circulation*.

[36] Heath MF, Costa-Jussa FR, Jacobs JM, Jacobson W (1985). The induction of pulmonary phospholipidosis and the inhibition of lysosomal phospholipases by amiodarone. *British Journal of Experimental Pathology*.

[37] Luallmann-Rauch R, Reil GH (1974). Chlorphentermine-induced lipidosis-like ultrastructural alterations in lungs and adrenal glands of several species. *Toxicology and Applied Pharmacology*.

[38] Pirovino M, Muller O, Zysset T, Honegger U (1988). Amiodarone-induced hepatic phospholipidosis: correlation of morphological and biochemical findings in an animal model. *Hepatology*.

[39] Yagupsky P, Gazala E, Sofer S (1985). Fatal hepatic failure and encephalopathy associated with amiodarone therapy. *Journal of Pediatrics*.

[40] Vereckei A, Fehér E, György I (1991). Possibilities of antioxidant therapy in the prevention of side effects of amiodarone. *Orvosi Hetilap*.

[41] Breuer HWM, Bossek W, Haferland C, Schmidt M, Neumann H, Gruszka J (1998). Amiodarone-induced severe hepatitis mediated by immunological mechanisms. *International Journal of Clinical Pharmacology and Therapeutics*.

[42] Kaufmann P, Török M, Hänni A, Roberts P, Gasser R, Krähenbühl S (2005). Mechanisms of benzarone and benzbromarone-induced hepatic toxicity. *Hepatology*.

[43] Fromenty B, Fisch C, Labbe G (1990). Amiodarone inhibits the mitochondrial *β*-oxidation and fatty acids and produces microvesicular steatosis of the liver in mice. *Journal of Pharmacology and Experimental Therapeutics*.

[44] Fromenty B, Fisch C, Berson A, Letteron P, Larrey D, Pessayre D (1990). Dual effect of amiodarone on mitochondrial respiration. Initial protonophoric uncoupling effect followed by inhibition of the respiratory chain at the levels of complex I and complex II. *Journal of Pharmacology and Experimental Therapeutics*.

[45] Pollak PT (2010). How toxic is amiodarone to the liver?. *Journal of Gastrointestinal and Liver Diseases*.

[46] Yap SH, Rijntjes PJM, Moshage HJ, Croes H, Jap PHK (1987). Amiodarone-induced lysosomal inclusions in primary cultures of human hepatocytes. *Gastroenterology*.

[47] Kojima S, Kojima S, Ueno H, Takeya M, Ogawa H (2009). Increased density of the liver and amiodarone associated phospholipidosis. *Cardiol Res Pract*.

[48] Llanos L, Moreu R, Peiró AM (2009). Causality assessment of liver injury after chronic oral amiodarone intake. *Pharmacoepidemiology and Drug Safety*.

[49] Gill J, Heel RC, Fitton A (1992). Amiodarone. An overview of its pharmacological properties, and review of its therapeutic use in cardiac arrhythmias. *Drugs*.

[50] Adams PC, Bennett MK, Holt DW (1986). Hepatic effects of amiodarone. *British Journal of Clinical Practice. Supplement*.

[51] Simon JB, Manley PN, Brien JF, Armstrong PW (1984). Amiodarone hepatotoxicity simulating alcoholic liver disease. *The New England Journal of Medicine*.

[52] Brien JF, Jimmo S, Brennan FJ (1987). Distribution of amiodarone and its metabolite, desethylamiodarone, in human tissues. *Canadian Journal of Physiology and Pharmacology*.

[53] Lettéron P, Sutton A, Mansouri A, Fromenty B, Pessayre D (2003). Inhibition of microsomal triglyceride transfer protein: another mechanism for drug-induced steatosis in mice. *Hepatology*.

[54] Gluck N, Fried M, Porat R (2011). Acute amiodarone liver toxicity likely due to Ischemic hepatitis. *Israel Medical Association Journal*.

[55] Klotz U (2007). Antiarrhythmics: elimination and dosage considerations in hepatic impairment. *Clinical Pharmacokinetics*.

[56] Pratt DS, Kaplan MM (2000). Evaluation of abnormal liver-enzyme results in asymptomatic patients. *The New England Journal of Medicine*.

[57] Mattar W, Juliar B, Gradus-Pizlo I, Kwo PY (2009). Amiodarone hepatotoxicity in the context of the metabolic syndrome and right-sided heart failure. *Journal of Gastrointestinal and Liver Diseases*.

[58] Babatin M, Lee SS, Pollak PT (2008). Amiodarone hepatotoxicity. *Current Vascular Pharmacology*.

[59] Tisdale JE, Follin SL, Ordelova A, Webb CR (1995). Risk factors for the development of specific noncardiovascular adverse effects associated with amiodarone. *Journal of Clinical Pharmacology*.

[60] Kum LCC, Chan WWL, Hui HHY (2006). Prevalence of amiodarone-related hepatotoxicity in 720 Chinese patients with or without baseline liver dysfunction. *Clinical Cardiology*.

[61] Rätz Bravo AE, Drewe J, Schlienger RG, Krähenbühl S, Pargger H, Ummenhofer W (2005). Hepatotoxicity during rapid intravenous loading with amiodarone: description of three cases and review of the literature. *Critical Care Medicine*.

[62] Pollak PT, Shafer SL (2004). Use of population modeling to define rational monitoring of amiodarone hepatic effects. *Clinical Pharmacology and Therapeutics*.

[63] Burgess C, Blaikie A, Ingham T, Robinson G, Narasimhan S (2006). Monitoring the use of amiodarone: compliance with guidelines. *Internal Medicine Journal*.

[64] Pollak PT, You YD (2003). Monitoring of hepatic function during amiodarone therapy. *American Journal of Cardiology*.

[65] Pollak PT, Sharma AD, Carruthers SG (1990). Relation of amiodarone hepatic and pulmonary toxicity to serum drug concentrations and superoxide dismutase activity. *American Journal of Cardiology*.

[66] Podrid PJ (1995). Amiodarone: reevaluation of an old drug. *Annals of Internal Medicine*.

